# Diagnostic and Prognostic Value of Circulating CircRNAs in Cancer

**DOI:** 10.3389/fmed.2021.649383

**Published:** 2021-03-18

**Authors:** Mina Wang, Feiyu Xie, Jiaran Lin, Yihan Zhao, Qian Zhang, Zehuan Liao, Peng Wei

**Affiliations:** ^1^School of Traditional Chinese Medicine, Beijing University of Chinese Medicine, Beijing, China; ^2^Department of Acupuncture and Moxibustion, Beijing Hospital of Traditional Chinese Medicine, Capital Medical University, Beijing Key Laboratory of Acupuncture Neuromodulation, Beijing, China; ^3^Oncology Department, Wangjing Hospital of China Academy of Chinese Medical Sciences, Beijing, China; ^4^Department of Nephrology and Endocrinology, Dongzhimen Hospital Affiliated to Beijing University of Chinese Medicine, Beijing, China; ^5^National Clinical Research Center for Chinese Medicine Cardiology, Xiyuan Hospital, China Academy of Chinese Medical Sciences, Beijing, China; ^6^School of Biological Sciences, Nanyang Technological University, Singapore, Singapore; ^7^Department of Microbiology, Tumor, and Cell Biology (MTC), Karolinska Institutet, Biomedicum, Solnavägen, Sweden

**Keywords:** circRNA, circulation, cancer diagnosis, cancer prognosis, biomarker

## Abstract

Cancer has been regarded as one of the leading causes of mortality worldwide. Diagnostic and prognostic biomarkers with high sensitivity and specificity for cancer play a crucial role in preventing or treating cancer. Circular RNAs (circRNAs), which hold great potential for the management of cancer patients due to their abundance, stable property, and high specificity in serum, plasma, and other body fluids, can be used as non-invasive and blood-based biomarkers in cancer diagnosis and prognosis. There are four types of circRNAs including exonic circRNAs (ecircRNA), intronic circRNAs, exon-intron circRNAs (EIciRNA), and intergenic circRNAs. CircRNAs can act as miRNA sponges, affect protein translation, interplay with RNA binding proteins, regulate protein recruitment, and modulate protein scaffolding and assembly. Therefore, the multifunctionalities of circRNAs make them ideal for detecting and predicting cancer. Indeed, circRNAs manifest high sensitivity and specificity in more than ten types of cancer. This review aims to consolidate the types and functions of circRNAs, as well as discuss the diagnostic and prognostic value of circulating circRNAs in cancer.

## Introduction

Cancer, which was estimated to lead to 18.1 million new cases and 9.6 million deaths according to GLOBOCAN 2018, is regarded as one of the most detrimental disease to our society (1–6). Given the concealment and non-specificity of clinical manifestations, most cancer cases are diagnosed at advanced stages. Hence the best therapies are only suitable for limited number of patients, let alone frequent dug resistance and adverse events (7–10). Taking tissue biopsies and imaging examinations as well as using cancer biomarkers are some of the most widely used methods in cancer diagnosis. However, these methods have their own limitations. For example, although taking tissue biopsies is viewed as the gold standard of cancer diagnosis, there is a risk for patients with coagulation dysfunction or other concomitant diseases to take biopsies (11). Moreover, only tumors growing to certain size can be detected by imaging examination such as CT (12). Also, many cancer biomarkers are unable to possess both superior sensitivity and specificity simultaneously. Therefore, it is an urgent need to discover effective cancer biomarkers for people to prevent or to receive treatment at early stages of cancer.

With progress achieved in the field of non-coding RNA (ncRNA), circular RNAs (circRNAs), a unique type of ncRNA characterized by its loop structure, have emerged as playing a significant role in carcinogenesis, metastasis, recurrence and multidrug resistance (13). CircRNAs are formed through back-splicing process whereby a downstream 5′ splice donor site is ligated with an upstream 3′ splice acceptor site to form a single-strand covalently closed loop. Afterwards, the spliceosome removes all or part of introns and the remaining sequences are linked together (14). CircRNAs are of considerable diagnostic and prognostic value not only due to the convenience for detection but also the exhibition of tissue/developmental-stage-specific expression. CircRNAs are prone to be detected because of large quantity, for example, over 25,000 different circRNAs having been identified in human fibroblasts (15). In addition, alternative circularization caused by the competition between different flanking complementary introns increases the isoforms of circRNAs, thus contributing to the abundance of circRNAs (16). Besides, the covalently closed loop structure without 5′-3′ polarity or polyadenylated tail confers great stability upon circRNAs, preventing them from being degraded by RNase R, debranching enzyme or RNA exonuclease (17). As a result, the average half-life of circRNAs in cells is much longer than that of mRNA (messenger RNA). Furthermore, Rybak-Wolf et al. (18) analyzed thousands of neuronal human and mouse circRNAs, discovering that circRNAs are highly conserved in sequence. This phenomenon might result from the conservation of splicing regulatory elements, complementary flanking introns and few polymorphisms of miRNA target sites of circRNA sponges (19). Accoring to Memczak et al., (20) circRNAs are much higher expressed in peripheral whole blood compared with linear ncRNAs, and consequently circRNAs can be detected without using invasive methods. Last but not least, tissue/developmental-stage-specifc expression pattern is a distinguished feature of circRNAs, which means their expression levels are extremely diverse and variable based on cell types and development stage of tissues, respectively (21). With advantages elucidated above, circRNAs are practical biomarkers in cancer diagnosis and prognosis.

In this review, we will focus on sorting out the types and functions of circRNAs and the expression levels of circulating circRNAs systematically according to the latest research. Moreover, perspectives and challenges will be proposed to analyze the application prospect of circRNAs in cancer diagnosis and prognosis.

## Types and Functions of CircRNAs

**Figure 1:**
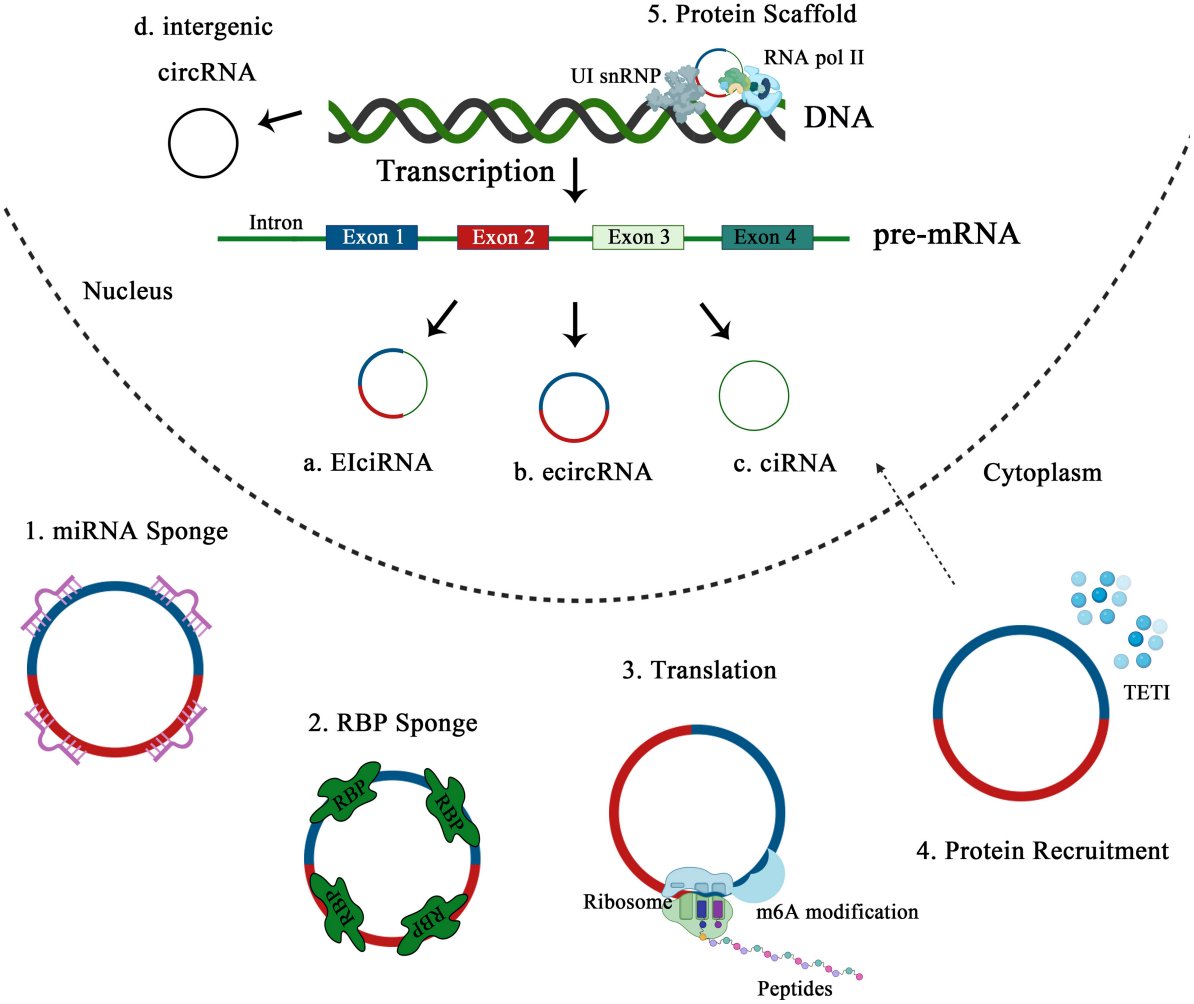
Schematic diagram of the biogenesis and functions of circRNA.

In recent years, ncRNAs have been recognized as a new sensitive, non-invasive biomarker for diagnosis, prognosis and even prediction to therapeutic responses due to the high stability in body fluids (urine, plasma, exosomes, etc.) and the development of detection techniques (22), among which circRNAs are presumably more stable than most linear RNAs because they form a unique, circular, covalently closed continuous loop that is resistant to exonuclease-mediated degradation because it has no 5′ or 3′ ends (Figure 1).

### Types of CircRNAs

CircRNAs are divided into four categories: exonic circRNAs (ecircRNA), intronic circRNAs, exon-intron circRNAs (EIciRNA), and intergenic circRNAs (23). Most circRNAs come from exons of protein coding genes via “back-splicing” (24), where a downstream splice donor site (5′ splice site) connects to an upstream acceptor splice site (3′ splice site). There are three possible models of ecircRNAs biogenesis: lariat-driven circularization, intron-pairing-driven circularization, and resplicing-driven circularization (25). Similarly formed like ecircRNAs, EIciRNAs are circularized with introns “retained” between exons, which predominantly localize in the nucleus and are associated with RNA polymerase II in human cells (26). CircRNAs from introns include circular intronic RNAs (ciRNAs), excised group I introns, excised group II introns, intron lariats, and excised tRNA introns (27). GU-rich sequences near the 5′ splice site and C-rich sequences near the branch point are sufficient for an intron to escape debranching and become a stable circRNA (28). Sequence analyses indicated that intergenic or intronic circRNAs generally showed only weak conservation, whereas coding exons serve additional, presumptively regulatory functions when expressed within circRNAs (29). Generally, knowledge about these circularized transcripts, and the specific mechanism regulating the biogenesis of circRNAs is still lacking.

### Functions of CircRNAs

#### CircRNAs Act as miRNA Sponges

MicroRNAs (miRNAs) are important post-transcriptional regulators of gene expression that act by direct base pairing to target sites within untranslated regions of mRNAs. A few years ago, two independent studies discovered that endogenous circRNAs can work as miRNA sponges. This means that circRNAs bind to miRNAs and consequently repress their function (29, 30). For example, ciRS-7 (also termed as CDR1as), acting as a miR-7 sponge, contains more than 70 selectively conserved miRNA target sites, and is highly and widely associated with Argonaute (AGO) proteins in a miR-7-dependent manner (30). Expression of ciRS-7 inhibits miR-7 activity that leads to increase in expression of miR-7 targets such as UBE2A, EGFR, PI3K, etc. (30–33). The sex-determining region Y (SRY) gene produces a testis-specific circRNA known as cir-SRY. Studies have shown that cir-SRY serves as a miR-138 sponge and 16 putative target sites have been identified (30). Unlike the two circRNAs mentioned above that contain only one kind of miRNA binding site, circHIPK3 could bind to multiple miRNAs (9 miRNAs with 18 binding sites), suggesting that one circRNA might be associated with a variety of miRNAs. CircHIPK3 could regulate cell growth by sponging various miRNAs especially miR-124 in human cells (34).

#### Translating Proteins

Although circRNAs generally do not translate proteins due to the lack of 5′ cap structure for translation initiation, some circRNAs can be internally modified by m6A and start translating small peptides (35). The small peptide can be bioactive such as a new 21-kDa protein encoded by circ-FBXW7, which is termed FBXW7-185aa. Upregulation of FBXW7-185aa in cancer cells could repress proliferation and cell cycle acceleration, while knockdown of FBXW7-185aa could lead to cancerous phenotypes *in vitro* and *in vivo*. CircPINTexon2 encodes PINT87aa which is decreased in glioma tissues and negatively impacts the clinical prognosis of glioma. Furthermore, PINT87aa could bind to the polymerase-associated factor 1 (PAF1) complex to prohibit the RNA elongation of multiple oncogenes (36). Circular SHPRH (circ-SHPRH) uses overlapping genetic codes to form a “UGA” stop codon, encoding a novel 17 kDa protein named after SHPRH-146aa. Both circ-SHPRH and SHPRH-146aa are amply expressed in normal human brains and decreased in glioblastoma. The overexpression of SHPRH-146aa in U251 and U373 glioblastoma cells weakens their malignant behavior and tumorigenicity *in vitro* and *in vivo* (37).

#### Interaction With RNA Binding Proteins

CircRNAs can also bind to RNA binding proteins (RBP) as protein sponges. Ashwal-Fluss et al. (38) observed that the splicing factor Muscleblind (MBL) strongly and specifically binds to circMbl, the circRNA generated from its own RNA. When the MBL protein is redundant, circMbl could sponge out the extra proteins by binding to it. Du et al. (39) reported that ectopic expression of circ-Foxo3 suppressed cell cycle progression by binding to the cell cycle proteins cyclin-dependent kinase 2 (also known as cell division protein kinase 2 or CDK2) and cyclin-dependent kinase inhibitor 1 (or p21), forming the structure of a ternary complex.

#### Protein Scaffolding and Assembly

In contrast to the protein sponge model, circRNAs also function as dynamic protein scaffolds enabling connection and assembly of proteins. Li et al. (26) revealed that EIciRNA could promote RNA Polymerase II-mediated parental gene transcription by acting as a linker of U1 snRNP and the pol II complex. Circ-Amotl1 has also been reported to serve as a protein scaffold by binding to AKT and PDK1 to form ternary complexes, hence promoting the nuclear translocation of pAKT (40).

#### Protein Recruitment

CircRNAs can also tether spliceosome for splicing or target chromatin modifiers to particular sites to modulate gene expression. For instance, FLI1 circular RNA termed with FECR1 could collaborate with the FLI1 promoter. FECR1 binds to the FLI1 promoter in cis and recruits Ten-Eleven Translocation methylcytosine dioxygenase 1 (TET1) demethylase, which may regulate metastasis of breast cancer cells by coordinating DNA methylation and demethylation (41).

## Circulating circRNAs in Different Cancers

CirRNAs are abundant and stable molecules with high cell specificity presenting in serum, plasma and other body fluids, which can be used as non-invasive and blood-based biomarkers in cancer diagnosis and prognosis (42) (Refer to Table 1).

**Table 1:** Circulating circRNAs in different types of cancer.

**Cancer type**	**Name of circRNAs**	**Changes**	**Functions**	**Sample**	**ROC curve**	**References**
NPC	circRNA_000285	Upregulated	Diagnosis and prognosis	Serum	N/A	(34)
	circMYC	Upregulated	Diagnosis and prognosis	Plasma	AUC: 0.945 Sensitivity: 90.24% Specificity: 94.51%	(35)
	Has_circ_0066755	Upregulated	Diagnosis	Plasma	AUC: 0.904	(36)
ESCC	circGSK3β	Upregulated	Diagnosis and prognosis	Plasma	AUC: 0.782 Sensitivity: 86.1% Specificity: 58.1%	(38)
	circRNA_0004771	Upregulated	Diagnosis and prognosis	Plamsa	N/A	(39)
	circ-SLC7A5	Upregulated	Diagnosis and prognosis	Plasma	AUC: 0.772	(40)
SCLC	FECR1	Upregulated	Diagnosis and prognosis	Serum	N/A	(43)
NSCLC	circFARSA	Upregualted	Diagnosis	Plasma	AUC: 0.710	(44)
	F-circEA	Upregualted	Diagnosis and prognosis	Plasma	N/A	(45)
	hsa_circ_0005962	Upregualted	Diagnosis	Plasma	AUC: 0.730 Sensitivity: 71.9% Specificity: 72.2%	(46)
	hsa_circ_0086414	Downregulated	Diagnosis	Plasma	AUC: 0.780 Sensitivity: 77.1% Specificity: 66.7%	(46)
Breast cancer	hsa_circ_0001785	Upregualted	Diagnosis and prognosis	Plasma	AUC: 0.771 Sensitivity: 78.6% Specificity: 75.6%	(47)
Gastric cancer	hsa_circ_002059	Downregulated	Diagnosis and prognosis	Plasma	AUC: 0.730 Sensitivity: 81.0% Specificity: 62.0%	(48)
	hsa_circ_0000190	Downregulated	Diagnosis	Plasma	AUC: 0.060 Sensitivity: 41.4% Specificity: 87.5%	(49)
	hsa_circ_0000181	Upregulated	Diagnosis and prognosis	Plasma	AUC: 0.582 Sensitivity: 20.6% Specificity: 99.0%	(50)
	hsa_circ_0001649	Downregulated	Diagnosis	Serum	AUC: 0.834 Sensitivity: 71.1% Specificity: 81.6%	(51)
	hsa_circ_0000745	Downregulated	Diagnosis	Plasma	AUC: 0.683 Sensitivity: 85.5% Specificity: 45.0%	(52)
	circ-SFMBT2	Upregulated	Diagnosis	Plasma	AUC: 0.759 Sensitivity: 80.6% Specificity: 63.9%	(53)
	circ-ERBB2	Upregulated	Prognosis	Plasma	N/A	(54)
HCC	hsa_circ_0001445	Downregulated	Diagnosis	Plasma	AUC: 0.862 Sensitivity: 94.2% Specificity: 71.2%	(55)
	hsa_circ_104075	Upregulated	Diagnosis	Serum	AUC: 0.973 Sensitivity: 96.0% Specificity: 98.3%	(56)
	circ_0009582	Upregulated	Diagnosis	Plasma	AUC: 0.805	(57)
	circ_0037120	Upregulated	Diagnosis	Plasma	AUC: 0.835	(57)
	circ_0140117	Upregulated	Diagnosis	Plasma	AUC: 0.845	(57)
Pancreatic cancer	circ-LDLRAD3	Upregulated	Diagnosis and prognosis	Plasma	AUC: 0.670 Sensitivity: 57.4% Specificity: 70.5%	(58)
	circ-PDE8A	Upregulated	Prognosis	Plasma	N/A	(59)
	circ-IARS	Upregulated	Prognosis	Plasma	N/A	(60)
CRC	hsa_circ_0007534	Upregulated	Diagnosis and Prognosis	Plasma	AUC: 0.780 Sensitivity: 92.0% Specificity: 52.2%	(61)
	hsa_circ_0001649	Upregulated	Diagnosis	Serum	N/A	(62)
	hsa_circ_0004585	Upregulated	Diagnosis	Peripheral blood	N/A	(63)
	circ-CCDC66, circ-ABCC1, circ-STIL	Downregulated	Diagnosis	Plasma	AUC: 0.780 Sensitivity: 64.4% Specificity: 85.2%	(64)
	circVAPA	Upregulated	Diagnosis and prognosis	Plasma	AUC: 0.724	(65)
	hsa_circ_0000370	Upregulated	Diagnosis and prognosis	Plasma	AUC: 0.815	(66)
	hsa_circ_0082182	Upregulated	Diagnosis and prognosis	Plasma	AUC: 0.737	(66)
	hsa_circ_0035445	Downregulated	Diagnosis and prognosis	Plasma	AUC: 0.703	(66)
	hsa_circ_0004771	Upregulated	Diagnosis	Serum	AUC: 0.880	(67)
	hsa_circ_0002320	Downregulated	Diagnosis and prognosis	Plasma	AUC: 0.823	(68)
Bladder cancer	hsa_circ_0003221	Upregulated	Prognosis	Whole blood	N/A	(69)
	circFARSA	Upregulated	Diagnosis and prognosis	Serum	N/A	(70)
	circSHKBP1	Upregulated	Diagnosis and Prognosis	Serum	AUC: 0.804	(70)
	circBANP	Upregulated	Prognosis	Serum	N/A	(70)
	hsa_circ_0000285	Downregulated	Prognosis	Serum	N/A	(71)
Endometrial cancer	hsa_circ_0109046	Upregulated	Prognosis	Serum	N/A	(72)
	hsa_circ_0002577	Upregulated	Prognosis	Serum	N/A	(72)
Cervical cancer	hsa_circ_0101996	Upregulated	Diagnosis	Whole blood	AUC: 0.906	(73)
	hsa_circ_0101119	Upregulated	Diagnosis	Whole blood	AUC: 0.887	(73)
	circFoxO3a	Downregulated	Prognosis	Serum	N/A	(74)
Leukemia	circBA9.3	Upregulated	Prognosis	Whole blood	N/A	(75)
	circHIPK3	Upregulated	Prognosis	Serum	N/A	(76)
Melanoma	hsa_circ_0001591	Upregulated	Prognosis	Serum	N/A	(77)
Osteosarcoma	hsa_circ_0081001	Upregulated	Diagnosis and prognosis	Serum	AUC: 0.898	(78)
	hsa_circ_0000885	Upregulated	Diagnosis and prognosis	Serum	AUC: 0.783	(79)

*NPC, nasopharyngeal carcinoma; ESCC, esophageal squamous cell carcinoma; SCLC, small-cell lung cancer; NSCLC, non-small-cell-lung cancer; HCC, hepatocellular carcinoma; CRC, colorectal cancer; N/A, not available; AUC, area under the curve*.

### Nasopharyngeal Carcinoma

Nasopharyngeal carcinoma (NPC) as a highly invasive and metastatic head and neck malignant tumor, occurs commonly in Southern China and Southeast Asia. Plasma Epstein-Barr virus (EBV) DNA has been reported to be used for population screening (43), whereas several circRNAs have showed effect in diagnosis and prognosis of NPC. Shuai et al. (44) found that circRNA_000285 was significantly increased in the serum samples of patients with NPC, in comparison with that of healthy controls. Notably, the level of circRNA_000285 was upregulated by 3-fold in NPC patients with radiation resistance compared with those were radiation sensitive. Also, patients with high expression of circRNA_000285 had poorer overall survival rate than those with low expression. Besides, circMYC was highly expressed in patients with NPC as well, and the expression was significantly correlated with tumor size, lymphatic metastasis, tumor-node- metastasis stage (TNM stage), survival rate and disease recurrence. The AUC (area under the curve) value for differentiating radioresistant patients from radiosensitive patients was 0.945, with a sensitivity of 90.24% and a specificity of 94.51% (45). Wang et al. (46) observed a significant increase of hsa_circ_0066755 in the NPC patients' plasma with an AUC value of 0.904. Meanwhile, plasma hsa_circ_0066755 testing presented a comparable diagnostic accuracy to the magnetic resonance imaging (MRI) (46). Thus, circRNA_000285 and circMYC represent diagnostic and prognostic value for NPC, while hsa_circ_0066755 exhibits merely diagnostic characteristic.

### Esophageal Cancer

Esophageal cancer is considered as one of the most aggressive and lethal malignant tumors with a poor 5-year survival rate due to inefficient diagnosis methods. There are two major type of esophageal cancer: esophageal adenocarcinoma (EAC) and esophageal squamous cell carcinoma (ESCC). Classical biomarkers include serum Squamous Cell Carcinoma Antigen (SCCA), Carbohydrate antigen-19-9 (CA19-9), and Carcinoma Embryonic Antigen (CEA). However, the lack of sensitivity and specificity limits them to be effectively used in the early detection of esophageal cancer. More and more evidence has indicated that dysregulation of circRNAs functions in the progression of various cancers, including esophageal cancer (80).

Recently, Hu et al. (2) found that the level of circGSK3β was significantly increased in the plasma of ESCC patients compared to that of healthy people. According to ROC (receiver operating characteristic) analysis, circGSK3β presented a sensitivity of 86.1%, a specificity of 58.1%, and an AUC of 0.782 in distinguishing ESCC patients from healthy people, indicating that plasma circGSK3β has significantly diagnostic value. Moreover, the AUC for circGSK3β in ESCC patients at early stages and healthy controls reached 0.793, with a sensitivity of 68.8% and a specificity of 81.3%. The diagnostic ability was verified in an independent validation cohort as well. The AUC of circGSK3β for ESCC/early stages of ESCC and healthy controls were 0.801 and 0.826, respectively. Additionally, the study has shown that the plasma level of circGSK3β was reduced in 75% of ESCC patients after surgery, while that of circGSK3β was increased in ESCC patients with recurrence/metastasis 10 months after surgery, suggesting the plasma level of circGSK3β has predictive values for clinical improvement. Besides, Huang et al. (81) demonstrated an upregulation of hsa_circ_0004771 in plasma was associated with a heavier tumor burden and a poor prognosis, causing a lower overall survival. The origin of hsa_circ_0004771 in plasma may be tumor-derived exosomes, and the ROC analysis revealed effective diagnostic performance of hsa_circ_0004771. Wang et al. discovered that plasma circ-SLC7A5 could serve as novel diagnostic biomarker for ESCC detection, with the area under the ROC curve of 0.772, and plasma circ-SLC7A5 expression was significantly up-regulated in ESCC patients, positively related to TNM stage and tend to have a poor overall survival (47). These findings have confirmed the significant diagnostic and prognostic value of circGSK3β, hsa_circ_0004771, and circ-SLC7A5 in esophageal cancer.

### Lung Cancer

Lung cancer remains as the leading cause of cancer-related death worldwide, with more than 1.8 million new cases and nearly 1.6 million deaths estimated in 2012. Non-small-cell lung cancer (NSCLC) accounts for ~80–85% of all cases, and small cell lung cancer (SCLC), accounts for about 15% (54). To date, the diagnosis for lung cancer include tumor-liberated proteins (e.g., CEA, NSE, TPA, chromogranin, CA125, CA19-9, and CYFRA21-1) and computed tomography (CT) (48). However, most patients are still diagnosed at advanced stages. Thus, having accurate and sensitive biomarkers is crucial for lung cancer diagnosis and prognosis.

Circulating circRNA emerges as a promising method for diagnosis and prognosis of lung cancer. Li et al. (49) compared the levels of serum FECR1 in SCLC patients with that in normal controls, they proved that FECR1 expression was higher in patients with SCLC, and much higher in those with distant metastasis. Notably, the level of serum FECR1 was strongly associated with chemotherapy response in SCLC patients, suggesting that serum FECR1 might be useful in detecting and tracking SCLC. As for NSCLC, the abundance of circFARSA in plasma has been reported to elevate in NSCLC patients, and the AUC was 0.710, which represents a potential diagnostic performance of circFARSA for NSCLC (50). Meanwhile, F-circEA specifically elevates in EML4-ALK fusion gene positive NSCLC patients' plasma, thus monitoring the EML4-ALK translocation and guiding the EML4-ALK-targeted NSCLC therapy. Also, F-circEA can independently promote cancer cell migration and invasion, leading to tumor progression. Taken together, F-circEA could be a “liquid biopsy” biomarker to diagnose NSCLC, as well as provide clinical implications for NSCLC therapy (51). In addition, Liu et al. (52) observed significant upregulations of hsa_circ_0005962 and downregulations of hsa_circ_0086414 in lung adenocarcinoma plasma and cells, and the plasma level of hsa_circ_0005962 was decreased in postoperative patients compared with preoperative patients. According to ROC analysis, the AUC of hsa_circ_0005962 was 0.730, with a sensitivity of 71.9% and specificity of 72.2%, and the AUC of hsa_circ_0086414 was 0.780, with a sensitivity and specificity of 77.1 and 66.7%, respectively. The best discriminatory ability was shown in the combination of the two circRNAs, with with an AUC of 0.810, sensitivity of 77.8% and specificity of 72.2%. Therefore, for lung cancer, FECR1 and F-circEA have the capability of both diagnosis and prognosis, whereas, circFARSA, hsa_circ_0005962 and hsa_circ_0086414 could serve as non-invasive diagnostic biomarkers.

### Breast Cancer

Breast cancer is one of the most common malignant tumors for woman in the world. The neoplasm stage (when the disease is diagnosed) plays a key role in overall survival, with more than 95% of 5-year survival rate for early stage, but only 20% of that for distant metastasis patients. Although the diagnosis for breast cancer is developing, and conventional serum biomarkers like CEA, CA15-3, and CA125 have been used as ideal approaches for detecting breast cancer because of their low invasiveness (53, 82), high-efficiency and specific clinical molecules are still desired for the diagnosis and prognosis of breast cancer. Yin et al. (83) selected three candidate circRNAs (hsa_circ_0001785, hsa_circ_0108942 and hsa_circ_0068033) out of 41 dysregulated circRNAs in the plasma of breast cancer patients, and hsa_circ_0001785 exhibited the highest diagnostic value with an AUC of 0.771, and a sensitivity of 78.6% and a specificity of 75.6%, indicating hsa_circ_0001785 could be a promising biomarker for breast cancer detection. They also compared the diagnostic efficiency of hsa_circ_0001785 with CEA and CA15-3 using plasma samples from 57 breast cancer patients, and hsa_circ_0001785 had the highest AUC value (0.784) compared with CEA (0.562) and CA15-3 (0.629). Notably, an even higher AUC value (0.839) was found in the combination of these three molecules. Moreover, the sensitivity and specificity of hsa_circ_0001785 (sensitivity: 76.4%, specificity: 70.0%) were significantly higher than that of CEA (sensitivity: 43.1%, specificity: 51.4%) and CA15-3 (sensitivity: 50.5%, specificity: 57.9%), and the combination of hsa_circ_0001785, CEA and CA15-3 showed the highest diagnostic efficiency with a sensitivity and a specificity of 75.8 and 90.4%, respectively. Besides, the plasma expression of hsa_circ_0001785 was significantly associated with histological grade (*P* = 0.013), TNM stage (*P* = 0.008) and distant metastasis (*P* = 0.016), which can predict the progression of breast cancer. Also, a lower plasma level of hsa_circ_0001785 was shown in postoperative patients compared with preoperative patients. Thus, the recurrence of postoperative cancer patients can be monitored by the plasma level of hsa_circ_0001785. The results of this study have confirmed the diagnostic and prognostic value of plasma hsa_circ_0001785 in breast cancer.

### Gastric Cancer

Gastric cancer is the third leading cause of cancer-related death worldwide. CEA and CA19-9 are two non-invasive and highly practical circulating biomarkers that have been used clinically. However, they present low sensitivity and specificity for gastric cancer diagnosis (84). The levels of hsa_circ_002059 have been introduced to be lower in preoperative plasma samples than in postoperative plasma samples, and the AUC was 0.730, the sensitivity and specificity were 81.0 and 62.0%, respectively. Also, the low expression levels of hsa_circ_002059 were closely related to TNM stage and distal metastasis (55). Similarly, the plasma level of hsa_circ_0000190 was downregulated in the gastric cancer patients, with an AUC of 0.060, a sensitivity of 41.4%, and a specificity of 87.5%, when combining the use of tissue and plasma hsa_circ_0000190, the values of AUC, sensitivity and specificity were increased to 0.780, 71.2%, and 75.0%, respectively. However, it did not present any association with clinicopathological features such as age, gender, diameter, differentiation, lymphatic metastasis, distal metastasis, invasion, and TNM stage, but its level was higher in CEA positive patients (56). Same result was found in a study of hsa_circ_0000181 that CEA positive patients had higher plasma level of hsa_circ_0000181. Also, hsa_circ_0000181 was significantly decreased in gastric cancer plasma, and its reduced level was significantly associated differentiation. In addition, the AUC of hsa_circ_0000181 was 0.582, the specificity and sensitivity were 20.6 and 99.0%, respectively (57).

Besides, Li et al. (85) demonstrated that hsa_circ_0001649 may have a negative correlation with gastric cancer, since hsa_circ_0001649 expression in serum was significantly increased in the postoperative compared with the preoperative with an AUC of 0.834, a sensitivity of 71.1% and a specificity of 81.6%. Furthermore, a study of Huang et al. (86) has proven that the expression level of hsa_circ_0000745 in plasma was downregulated in gastric cancer patients, and its level correlated with TNM stage. The AUC of hsa_circ_0000745 in plasma was 0.683, and the sensitivity and specificity were 85.5 and 45.0%, respectively. While the combination of hsa_circ_0000745 and CEA represented increased values of AUC (0.775), sensitivity (80.0%), and specificity (63.3%). Apart from these down-regulated circRNAs, a circRNA named circ-SFMBT2 revealed an increased expression level in gastric cancer plasma samples compared with healthy people, suggesting it could be applied to detect gastric cancer (87). Moreover, high circERBB2 level in the plasma was correlated to poor prognosis for gastric cancer, with a low overall survival and a high rate of recurrence, which may associate with sponging some miRNAs and triggering tumor growth or metastasis. Notably, the increased plasma level of circERBB2 tended to be related to men (84). Since TNM stage, distal metastasis and differentiation are pivotal factors for evaluating the prognosis of gastric cancer, these seven molecules hsa_circ_0000190, hsa_circ_0001649, hsa_circ_0000745, and circ-SFMBT2 show a potential value as diagnostic biomarkers, circERBB2 could serve as a prognostic biomarker, and hsa_circ_002059 and hsa_circ_0000181 have promising performances in both diagnosis and prognosis of gastric cancer.

### Hepatocellular Carcinoma

Liver cancer presented the sixth incidence and third mortality in malignancies worldwide in 2018. Hepatocellular carcinoma (HCC) is the most common form of liver cancer, accounting for 75–85% of all cases (1). HCC normally develops in patient with cirrhotic livers, which mainly result from hepatitis B virus (HBV)/ hepatitis C virus (HCV) infection or alcohol-related liver disease. For decades, the α-fetoprotein (AFP) (58), α-fetoprotein-L3 (AFP-L3) (59), and des-carboxy-prothrombin (DCP) (60) have been used for HCC diagnosis. Nevertheless, owing to their poor sensitivity and specificity, the current diagnostic methods are far from being satisfactory. Zhang et al. (88) assessed the diagnostic potential of plasma hsa_circ_0001445, using samples from 104 HCC patients, 57 cirrhosis patients, 44 HBV patients, and 52 healthy controls, and plasma hsa_circ_0001445 levels were significantly lower in HCC patients than other three groups. Compared with healthy controls, the AUC value of HCC patients was 0.862, with a specificity and a sensitivity of 94.2 and 71.2%, respectively. Moreover, the plasma hsa_circ_0001445 levels in HCC patients were associated with serum AFP levels, and the efficiency of the combination in distinguishing HCC patients from patients of cirrhosis (AUC = 0.743), from patients of HBV (AUC = 0.877), or from healthy controls (AUC = 0.970) was higher compared to using plasma hsa_circ_0001445 levels or serum AFP levels alone. Thus, the level of plasma hsa_circ_0001445 could be used for HCC diagnosis. Furthermore, Zhang et al. (89) identified that hsa_circ_104075 was highly expression in HCC serum, and the AUC value of hsa_circ_104075 (0.973) was higher than that of lncRNA (DANCR: 0.851, HULC: 0.855, UCA1: 0.735), miRNA (miR-223: 0.818, miR-21: 0.782) and classical protein biomarkers (AFP: 0.750, AFP-L3: 0.766, DCP: 0.771), with a sensitivity of 96.0% and a specificity of 98.3%. In addition, three circRNAs including circ_0009582, circ_0037120, and circ_0140117 were confirmed to be upregulated in HCC patients' plasma. The AUC values of circ_0009582, circ_0037120, circ_0140117, combination of circRNAs, AFP, and combination of circRNAs and AFP were 0.805, 0.835, 0.845, 0.857, 0.803 and 0.955, respectively. The positive predictive value (PPV) and negative predictive value (NPV) were 95 and 95%, respectively when compared to healthy controls, while the PPV and NPV were 84 and 80%, respectively when distinguishing HCC from HBV infection, suggesting these three circRNAs are able to predict HCC from patients with HBV or healthy controls (90). The abovementioned circRNAs involving hsa_circ_0001445, hsa_circ_104075, circ_0009582, circ_0037120, and circ_0140117 have the capability of diagnosing HCC, whereas, they show no property in prognosing HCC.

### Pancreatic Cancer

The estimated number of deaths due to pancreatic cancer was 432,000 in 2019 (61), with a 5-year survival rate of 9.3% (62). This is partly owing to the rapid invasion and early metastasis of pancreatic cancer as well as late diagnostic capabilities. The regular approaches applied clinically to detect pancreatic cancer include biopsy, CT and CA19-9 (63), and the discovery of biomarkers for the diagnosis of pancreatic cancer remains a major challenge. A study has verified that circ-LDLRAD3 was up-regulated in plasma of pancreatic cancer patients compared with healthy controls, and the level of plasma circ-LDLRAD3 was strongly related to CA19-9, N classification, venous invasion, and lymphatic invasion (64). The AUC value, sensitivity, and specificity of circ-LDLRAD3 were 0.670, 57.4%, and 70.5%, respectively, while the combination of circ-LDLRAD3 and CA19-9 increased the diagnostic value, with corresponding values for AUC, sensitivity, and specificity were 0.870, 80.3%, and 93.6%, respectively. Li et al. (65) analyzed the circ-PDE8A expression in plasma of pancreatic ductal adenocarcinoma (PDAC) patients and found that high expression of circ-PDE8A was associated with duodenal invasion, vascular invasion, T factor or TNM stage, as well as low survival rate. Moreover, high expression of circ-PDE8A acted as an independent risk for overall survival, suggesting circ-PDE8A expression was correlated with prognosis in PDAC.

Circ-IARS also has been reported to be an independent risk factor for pancreatic cancer, and it was presented an upregulation in plasma derived from patients with metastatic pancreatic cancer. Circ-IARS expression was positively associated with tumor vessel invasion, liver metastasis, and TNM stage and negatively associated with postoperative survival time (66). Besides, Seimiya et al. (67) suggested that circPDAC RNA might serve as a potential biomarker for PDAC, however, it was only examined in one serum of PDAC patient. Therefore, circ-PDE8A and Circ-IARS may be useful biomarkers for pancreatic cancer detection, circ-LDLRAD3 might be applied in the diagnosis and prognosis of pancreatic cancer.

### Colorectal Cancer

Colorectal cancer (CRC) is one of the most frequent digestive tract cancers, and the second leading cause of cancer-related deaths worldwide (1). The regular approaches for detecting CRC include fecal occult blood testing, colonoscopy, stool DNA testing and conventional tumor biomarkers (e.g., CEA and CA19-9) (68, 91). Colonoscopy screening and subsequent pathological examinations are the criterion standard for the diagnosis of CRC, while due to the low adherence rates and reproducibility, the diagnosis of a lot of CRC patients has been delayed. The circRNAs have emerged as non-invasive biomarkers for CRC. Zhang et al. (69) found that hsa_circ_0007534 expression was significantly higher in CRC patients' plasma than in healthy controls, and the increased plasma level of hsa_circ_0007534 was associated with higher incidence of clinical classifications, metastatic phenotype, and poor differentiation in CRC patients. According to ROC analysis, compared with healthy controls, the AUC value was 0.780, the sensitivity was 92.0%, and specificity was 52.2%. According to Kaplan-Meier analysis, the high hsa_circ_0007534 expression group exhibited a significantly poorer prognosis than the low hsa_circ_0007534 expression group. Moreover, another circRNA called hsa_circ_0001649 was observed an upregulation expression in serum samples of CRC patients after surgery (70). The upregulation expression was also found in CRC patients peripheral blood of hsa_circ_0004585 (71). Furthermore, the plasma levels of three circRNAs (circ-CCDC66, circ-ABCC1, and circ-STIL) showed significantly reduction in CRC patients compared to in healthy volunteers. The AUC of the three-circRNA panel was 0.780, with 64.4% sensitivity and 85.2% specificity. The decreased plasma levels circ-CCDC66 and circ-ABCC1 were found to be associated with precursor lesions of CRC, including colon adenomas and adenomatous polyps, and circ-CCDC66 and circ-STIL expressions could be effectively applied in detecting early- stage CRC (92). Besides, Li et al. (72) evaluated the diagnostic and prognostic values of circVAPA for CRC, the results showed that circVAPA expression in the plasma was significantly increased in CRC patients compared to in healthy controls, with an AUC of 0.724, and circVAPA expression exhibited higher in stage III and IV patients than in stage I and II patients, indicating that the plasma level of circVAPA showed a positive trend with TNM stage progression.

In addition, a study has demonstrated that in CRC plasma, hsa_circ_0000370, and hsa_circ_0082182 were significantly upregulated, whereas hsa_circ_0035445 was downregulated (93). The AUC values of hsa_circ_0000370, hsa_circ_0082182, and hsa_circ_0035445 were 0.815, 0.737, and 0.703, respectively. The levels of hsa_circ_0000370 and hsa_circ_0082182 was closely related to lymph node metastasis, and the hsa_circ_0035445 expression was related to the TNM stage. Notably, hsa_circ_0000370 presented no significant difference between preoperative and postoperative stages, but hsa_circ_0082182 and hsa_circ_0035445 had a significant difference between these two stages. Pan et al. (73) verified the level of serum hsa-circ-0004771 was increased in CRC patients compared to in healthy people and patients with benign intestinal diseases (BIDs), the AUC values were 0.590, 0.860 and 0.880 to discriminate BIDs, stage I/ II CRC patients and CRC patients from healthy people. Also, the expression of hsa-circ-0004771 was decreased in the serum of postoperative CRC patients. Besides, the level of plasma hsa_circ_0002320 was significantly downregulated in CRC patients, with an AUC of 0.823. Based on Kaplan-Meier analysis, the expression level of hsa_circ_0002320 was significantly connected with overall survival time: low level of hsa_circ_0002320 presented a poor overall survival time compared to CRC patients with high level of hsa_circ_0002320. Thus, hsa_circ_0002320 could be used for CRC diagnosis and prognosis (74). According to the studies of circRNAs in CRC, hsa_circ_0001649, hsa_circ_0004585, hsa-circ-0004771, circ-CCDC66, circ-ABCC1, and circ-STIL are non-invasive diagnostic biomarkers for CRC, and hsa_circ_0007534, hsa_circ_0000370, hsa_circ_0082182 hsa_circ_0035445, hsa_circ_0002320 and circVAPA can be applied for CRC diagnosis and prognosis.

### Bladder Cancer

Bladder cancer is the most common occurring malignancy of urinary system, with an estimated 549,000 new cases and 200,000 deaths in 2018 (1). Cystoscopy and biopsy are used as the criterion standard for diagnosing bladder cancer and no reliable biomarker is available to replace these methods at present (94). However, owing to the invasiveness of cystoscopy and biopsy, patient compliance is poor. Therefore, promising non-invasive diagnostic and prognostic biomarkers for bladder cancer are urgently needed. Xu et al. (75) investigated the expression of hsa_circ_0003221 also called circPTK2 in the blood of patients with bladder cancer (75). The level of blood hsa_circ_0003221 was significantly high in the blood samples, with strong association with several clinicopathologic characteristics, involving poor differentiation, N2-N3 lymph node metastasis, and TNM stage. Another study has indicated that circFARSA, circSHKBP1, circBANP, and lncRNA urothelial carcinoma-associated 1 (UCA1) are correlated with the diagnosis and prognosis of bladder cancer (76). The expression of circFARSA, circSHKBP, and lncRNA UCA1 were significantly higher in the serum of bladder cancer patients than healthy people, but there was no significant difference of serum circBANP expression in bladder cancer patients compared with healthy people. The AUC value of circSHKBP1 and lncRNA UCA1 signature to discriminate patients with bladder cancer from controls was 0.804, and that of this signature was 0.870 with respect of low-grade tumors. Moreover, the expression of circFARSA, circSHKBP1, and circBANP increased in the recurrent bladder cancer patients in comparison with non-recurrent patients, the AUC of the combination of circFARSA and circBANP to distinguish tumor recurrence from those without was 0.737. Chi et al. (95) confirmed that sa_circ_0000285 was significantly reduced in bladder cancer serum in contrast to healthy controls, and it was related to tumor size, differentiation, lymph node metastasis, distant metastasis and TNM stage. Furthermore, hsa_circ_0000285 expression was lower in cisplatin-resistant bladder cancer patients when comparing with those who were cisplatin-sensitive, indicating its important role in bladder cancer chemo-sensitivity. Therefore, as for bladder cancer, hsa_circ_0003221, hsa_circ_0000285 and circBANP play crucial roles in the prognosis, and circFARSA, circSHKBP1 can be used in the both diagnosis and prognosis.

### Endometrial Cancer

The prevalence of endometrial cancer has been increased that about 320,000 new cases and 76,000 deaths were estimated in 2012 (77). A study has aimed to explore the roles of serum circRNAs in endometrial cancer, and has elucidated that hsa_circ_0109046 and hsa_circ_0002577 were higher in the serum of patients with endometrial cancer than that that of healthy controls. Also, these two stable circRNAs could provide information about the development, metastasis and prognosis of endometrial cancer (96).

### Cervical Cancer

Cervical cancer ranks as the fourth common malignant tumor in women, with an estimated 570,000 cases and 311,000 deaths in 2018 worldwide (1). Several invasive prognostic factors have been applied in clinical practice, such as International Federation of Gynecology and Obstetrics (FIGO) stage, lymph node metastasis, lymph-vascular space invasion (LVSI), and deep stromal infiltration (97). However, non-invasive approaches for detecting and predicting cervical cancer are needed. Wang et al. (78) identified that the expression of hsa_circ_0101996, hsa_circ_0104649, hsa_circ_0104443, and hsa_circ_0101119 in whole blood had similar trend compared with in tumor tissues. Among them, hsa_circ_01- 01996 and hsa_circ_0101119 were upregulated more than 4-fold in whole blood of cervical cancer patient in contrast to healthy controls. ROC analysis revealed that hsa_circ_0101996 and hsa_circ_0101119 could distinguish cervical cancer patients from healthy controls with AUC values of 0.906 and 0.887, respectively. Intriguingly, there was a markedly improved AUC value of 0.964 when combining hsa_circ_0101996 and hsa_circ_0101119, and the sensitivity and specificity reached to 94.3 and 87.3%, respectively. Tang et al. (79) validated that circFoxO3a expression was decreased in serum of cervical patients compared with healthy volunteers, and the low expression was associated with severe stromal invasion and positive lymph node metastasis. Meanwhile, the lower level of serum circFoxO3a led to poorer overall survival and recurrence-free survival, suggesting circFoxO3a is a non-invasive tool for predicting survival in cervical cancer patients. Based on these two studies, hsa_circ_0101996 and hsa_circ_0101119 are useful for diagnosing cervical cancer, while circFoxO3a affects the prognosis of cervical cancer.

### Leukemia

Leukemia is a group of life-threatening malignant disorders of the blood and bone marrow, which lack of convenient methods to detect (98). Pan et al. (99) confirmed that circBA9.3 was associated with prognosis and tyrosine kinase inhibitors (TKIs) resistance in patients with chronic myeloid leukemia (CML). TKIs are effective therapy for CML patients, while some patients who are not responsive to TKIs have elevated circBA9.3 expression in the blood. Moreover, the overexpression of circBA9.3 accelerates the proliferation and inhibits apoptosis of cancer cells. Besides, circHIPK3 was also upregulated in peripheral blood mononuclear cells (PBMC) and serum samples from CML compared with healthy controls, and high circHIPK3 predicted a poor outcome of CML patients and may be an independent prognostic factor for CML development. Knockdown of circHIPK3 prevented proliferation and triggered apoptosis of cancer cell (100), indicating circHIPK3 can participate in the prognosis of CML.

### Melanoma

Melanoma originates from the basal layer of epidermis, and its morbidity keep increasing worldwide (101). A study has determined an upregulation of hsa_circ_0001591 in serum of patients with melanoma compared with normal controls. Moreover, high expression of hsa_circ_0001591 was correlated with inferior overall survival and disease-free survival in comparison with low expression group. The underlying mechanism may be related to the suppression of miR-431-5p (102).

### Osteosarcoma

Osteosarcoma is the most frequent primary malignant bone tumor in children, adolescents and young adults (103). Alkaline phosphatase (ALP) and lactate dehydrogenase (LDH) remain as the most common serum biomarkers used for diagnosing osteosarcoma in spite of their unsatisfactory sensitivity and specificity (104). A study identified that a circRNA named hsa_circ_0081001 was significantly increased in the serum of osteosarcoma patients, and the expression of hsa_circ_0081001 was gradually increased in the benign bone tumor and osteosarcoma compared to the control group (105). The AUC value of hsa_circ_0081001 (0.898) was higher than that of ALP (0.673) and LDH (0.800). Further analysis suggested that high hsa_circ_0081001 expression was associated with chemoresistant, lung metastasis or recurrence of osteosarcoma, and the expression was remarkably reduced in the serum after preoperative chemotherapy of two cycles and after operation. The results confirmed that serum hsa_circ_0081001 is an independent diagnostic and prognostic factor for osteosarcoma patients. Similar findings were demonstrated in another study that a significant increase of serum hsa_circ_0000885 was detected in osteosarcoma patients, especially in patients with Enneking stage IIB and III osteosarcoma, compared with early-stage osteosarcoma (106). Moreover, the levels of serum hsa_circ_0000885 were significantly higher in patients with osteosarcoma than patients with benign bone tumors or healthy controls. The AUC value for distinguishing between patients with osteosarcoma from healthy individuals was 0.783, while it was 0.714 when comparing osteosarcoma with benign bone tumors. Also, the increased level of serum hsa_circ_0000885 promoted lung metastasis and narrowed overall survival and disease-free survival, and the serum levels of hsa_circ_0000885 markedly decreased after chemotherapy and surgery. Thus, hsa_circ_0000885 could serve as a promising diagnostic and prognostic biomarker for osteosarcoma.

## Perspectives and Challenges

In comparison with linear RNAs, circRNAs have covalent circular structure without a 3′-end and 5′-end, which strengthens the characteristics of stability and abundance in circulating blood, especially in serum exosomes (107). Moreover, circRNAs are significantly upregulated or downregulated in various types of cancer with high AUC value, sensitivity, and specificity. Due to the biological functions of circRNAs and the non-invasive property of circulating circRNAs, relevant studies have verified that circulating circRNAs have the potential to act as promising biomarkers for diagnosing and prognosing different types of cancer. Nevertheless, there are several limitations when using circRNAs clinically to detect and predict cancer progression. A majority of circRNAs known to be dysregulated have been reported in only a single study with small scale of samples, and rare systematic validation studies with randomized trials based on these “verified” circRNAs with well-characterized and diverse patient samples have been conducted. Furthermore, a lack of standard naming system for circRNAs could cause confusion for further research. Also, since most circRNA sequences is shared with the miRNAs generated from the host gene, the identification, quantification, and validation, as well as overexpression and silencing strategies of circRNAs will be affected by specific back-splicing junction and become especially sensitive to biological and experimental artifacts (108). Besides, the underlying mechanisms have been scarcely reported in current studies. Therefore, studies based on larger samples and multiple centers should be conducted, and the “verified” circRNAs should be reconfirmed in more studies. Standard protocols including standard naming system, thorough validation, accurate quantification, careful confirmation, are urgently needed in order to compare findings from different studies. Additionally, we should investigate the role of circulating circRNAs in various types of cancer, whether they work as cancer promoting or suppressing agents, and how they work. A better understanding of the mechanisms of circulating circRNAs in cancer will promote the development of circRNA-based diagnostic tools and therapies for cancer. Although the study of circRNAs in cancer is still in its infancy, the current researches have validated the diagnostic and prognostic value of circulating circRNAs in different cancer types, and the importance given to circulating circRNAs as biomarkers for cancer diagnosis and prognosis has been increasing just over the last few years.

Apart from being effective biomarkers, economic benefit could be another important reason that emphasizes the value of circulating circRNAs. Since patients diagnosed with cancer are supposed to routinely monitor due to the risk of tumor recurrence and progression, the costs of cancer do tend to be high. According to the data from the Agency for Healthcare Research and Quality in the United States, there was a 98% increase from the agency projections in 2014 due to the high expenditure of cancer care and treatment which may approximately cost the American taxpayer 173 billion dollars in 2020 (109, 110). A significant portion of these costs is associated with the poor diagnosis of cancer. Studies have confirmed that incremental costs are significantly higher for advanced-stage cancer than for early-stage cancer (111), and rough estimate for cost-savings from early-stage cancer is 26 billion dollars (112). Thus, cancer biomarkers with high sensitivity and specificity can significantly reduce the cost. Circulating circRNAs as a kind of non-invasive cancer biomarkers with high sensitivity and specificity in different cancer, have been proven to be clinically cost-effective and treatment-effective because they can distinguish low-risk patients from high-risk patients at an early tumor stage (113). Therefore, the clinical generalization of circulating circRNAs in the future may save substantial expenditure in cancer care and treatment.

## Conclusion

Cancer remains as one of the leading causes of morbidity and mortality in the world, and circRNAs present a great capability to be promising biomarkers due to their high stability and conservation. Indeed, this discovery of the characteristics and biological functions of circRNAs has enabled a new understanding of the fundamental mechanisms of oncogenesis, and the relevant studies concerning circulating circRNAs has revealed exciting prospects for diagnosis and prognosis as cancer biomarkers. Although circulating circRNAs still lie in a new field with much to be explored, the source and function of circulating circRNAs should be investigated for better applying them in detecting and predicting cancer. As discussed in this review, there are still some challenges that should be overcome to further promote circulating circRNAs based biomarkers for clinical applications. By establishing this thorough review on circRNAs, we hope to provide valuable insights to how we can better tackle cancer in the future.

## Author Contributions

Conceptualization: ZL and PW. Writing: MW, FX, and JL. Figure and Table: YZ. Editing: MW, QZ, and ZL. Supervision: ZL and PW. Funding: PW. Figure 1 was created using Biorender.com. All authors contributed to the article and approved the submitted version.

## Conflict of Interest

The authors declare that the research was conducted in the absence of any commercial or financial relationships that could be construed as a potential conflict of interest.

## References

[1] Bray F Ferlay J Soerjomataram I Siegel RL Torre LA Jemal A. Global cancer statistics 2018: GLOBOCAN estimates of incidence and mortality worldwide for 36 cancers in 185 countries. CA Cancer J. Clin. (2018) 68:394–424. 10.3322/caac.21492 30207593

[2] Liao Z Tan ZW Zhu P Tan NS. Cancer-associated fibroblasts in tumor microenvironment–accomplices in tumor malignancy. Cell. Immunol. (2019) 343:103729. 10.1016/j.cellimm.2017.12.003 29397066

[3] Liao Z Wong SW Yeo HL Zhao Y. Smart nanocarriers for cancer treatment: clinical impact and safety. NanoImpact. (2020) 20:100253. 10.1016/j.impact.2020.100253

[4] Yang Y Yang T Liu S Cao Z Zhao Y Su X. Concentrated ambient PM2. 5 exposure affects mice sperm quality and testosterone biosynthesis. PeerJ. (2019) 7:e8109. 10.7717/peerj.8109 31799077 PMC6885350

[5] Wang M Yang Y Liao Z. Diabetes and cancer: epidemiological and biological links. World J. Diabetes. (2020) 11:227. 10.4239/wjd.v11.i6.227 32547697 PMC7284016

[6] Gonzalez-Molina J Gramolelli S Liao Z Carlson JW Ojala PM Lehti K. MMP14 in sarcoma: a regulator of tumor microenvironment communication in connective tissues. Cells. (2019) 8:991. 10.3390/cells8090991 31466240 PMC6770050

[7] Milman N Ginini L Gil Z. Exosomes and their role in tumorigenesis and anticancer drug resistance. Drug Resist. Updates. (2019) 45:1–12. 10.1016/j.drup.2019.07.003 31369918

[8] Liao Z Chua D Tan NS. Reactive oxygen species: a volatile driver of field cancerization and metastasis. Mol. Cancer. (2019) 18:1–10. 10.1186/s12943-019-0961-y 30927919 PMC6441160

[9] Wang M Tan Y Shi Y Wang X Liao Z Wei P. Diabetes and sarcopenic obesity: pathogenesis, diagnosis, and treatments. Front. Endocrinol. (2020) 11:568. 10.3389/fendo.2020.00568 32982969 PMC7477770

[10] Khan RMM Chua ZJY Tan JC Yang Y Liao Z Zhao Y. From pre-diabetes to diabetes: diagnosis, treatments and translational research. Medicina. (2019) 55:546. 10.3390/medicina55090546 31470636 PMC6780236

[11] Vaidyanathan R Soon RH Zhang P Jiang K Lim CT. Cancer diagnosis: from tumor to liquid biopsy and beyond. Lab Chip. (2019) 19:11–34. 10.1039/C8LC00684A 30480287

[12] Zaimy M Saffarzadeh N Mohammadi A Pourghadamyari H Izadi P Sarli A. New methods in the diagnosis of cancer and gene therapy of cancer based on nanoparticles. Cancer Gene Therapy. (2017) 24:233–43. 10.1038/cgt.2017.16 28574057

[13] Guarnerio J Bezzi M Jeong JC Paffenholz SV Berry K Naldini MM. Oncogenic role of fusion-circRNAs derived from cancer-associated chromosomal translocations. Cell. (2016) 165:289–302. 10.1016/j.cell.2016.03.020 27040497

[14] Li R Jiang J Shi H Qian H Zhang X Xu W. CircRNA: a rising star in gastric cancer. Cell. Mol. Life Sci. (2020) 77:1661–80. 10.1007/s00018-019-03345-5 31659415 PMC11104848

[15] Jeck WR Sorrentino JA Wang K Slevin MK Burd CE Liu J. Circular RNAs are abundant, conserved, and associated with ALU repeats. RNA. (2013) 19:141–57. 10.1261/rna.035667.112 23249747 PMC3543092

[16] Zhang XO Wang HB Zhang Y Lu X Chen LL Yang L. Complementary sequence-mediated exon circularization. Cell. (2014) 159:134–47. 10.1016/j.cell.2014.09.001 25242744

[17] Vicens Q Westhof E. Biogenesis of circular RNAs. Cell. (2014) 159:13–4. 10.1016/j.cell.2014.09.005 25259915

[18] Rybak-Wolf A Stottmeister C GlaŽar P Jens M Pino N Giusti S. Circular RNAs in the mammalian brain are highly abundant, conserved, and dynamically expressed. Mol. Cell. (2015) 58:870–85. 10.1016/j.molcel.2015.03.027 25921068

[19] Thomas LF Sætrom P. Circular RNAs are depleted of polymorphisms at microRNA binding sites. Bioinformatics. (2014) 30:2243–6. 10.1093/bioinformatics/btu257 24764460 PMC4207428

[20] Memczak S Papavasileiou P Peters O Rajewsky N. Identification and characterization of circular RNAs as a new class of putative biomarkers in human blood. PLoS ONE. (2015) 10:e0141214. 10.1371/journal.pone.0141214 26485708 PMC4617279

[21] Kristensen LS Andersen MS Stagsted LVW Ebbesen KK Hansen TB Kjems J. The biogenesis, biology and characterization of circular RNAs. Nat. Rev. Genet. (2019) 20:675–91. 10.1038/s41576-019-0158-7 31395983

[22] Martinez B Peplow PV. MicroRNAs in blood and cerebrospinal fluid as diagnostic biomarkers of multiple sclerosis and to monitor disease progression. Neural Regen. Res. (2020) 15:606–19. 10.4103/1673-5374.266905 31638082 PMC6975152

[23] Meng S Zhou H Feng Z Xu Z Tang Y Li P. CircRNA: functions and properties of a novel potential biomarker for cancer. Mol. Cancer. (2017) 16:1–8. 10.1186/s12943-017-0663-2 28535767 PMC5440908

[24] Suzuki H Zuo Y Wang J Zhang MQ Malhotra A Mayeda A. Characterization of RNase R-digested cellular RNA source that consists of lariat and circular RNAs from pre-mRNA splicing. Nucleic Acids Res. (2006) 34:e63. 10.1093/nar/gkl151 16682442 PMC1458517

[25] Chen I Chen CY Chuang TJ. Biogenesis, identification, and function of exonic circular RNAs. Wiley Interdiscipl. Rev. (2015) 6:563–79. 10.1002/wrna.1294 26230526 PMC5042038

[26] Li Z Huang C Bao C Chen L Lin M Wang X. Exon-intron circular RNAs regulate transcription in the nucleus. Nat. Struct. Mol. Biol. (2015) 22:256. 10.1038/nsmb.2959 25664725

[27] Lasda E Parker R. Circular RNAs: diversity of form and function. RNA. (2014) 20:1829–42. 10.1261/rna.047126.114 25404635 PMC4238349

[28] Wang F Nazarali AJ Ji S. Circular RNAs as potential biomarkers for cancer diagnosis and therapy. Am. J. Cancer Res. (2016) 6:1167. 27429839 PMC4937728

[29] Memczak S Jens M Elefsinioti A Torti F Krueger J Rybak A. Circular RNAs are a large class of animal RNAs with regulatory potency. Nature. (2013) 495:333–8. 10.1038/nature11928 23446348

[30] Hansen TB Jensen TI Clausen BH Bramsen JB Finsen B Damgaard CK. Natural RNA circles function as efficient microRNA sponges. Nature. (2013) 495:384–8. 10.1038/nature11993 23446346

[31] Kefas B Godlewski J Comeau L Li Y Abounader R Hawkinson M. microRNA-7 inhibits the epidermal growth factor receptor and the Akt pathway and is down-regulated in glioblastoma. Cancer Res. (2008) 68:3566–72. 10.1158/0008-5472.CAN-07-6639 18483236

[32] Pan H Li T Jiang Y Pan C Ding Y Huang Z. Overexpression of circular RNA ciRS-7 abrogates the tumor suppressive effect of miR-7 on gastric cancer via PTEN/PI3K/AKT signaling pathway. J. Cell. Biochem. (2018) 119:440–6. 10.1002/jcb.26201 28608528

[33] Lukiw W. Circular RNA (circRNA) in Alzheimer's disease (AD). Front. Genet. (2013) 4:307. 10.3389/fgene.2013.00307 24427167 PMC3875874

[34] Zheng Q Bao C Guo W Li S Chen J Chen B. Circular RNA profiling reveals an abundant circHIPK3 that regulates cell growth by sponging multiple miRNAs. Nat. Commun. (2016) 7:1–13. 10.1038/ncomms11215 27050392 PMC4823868

[35] Yang Y Fan X Mao M Song X Wu P Zhang Y. Extensive translation of circular RNAs driven by N 6-methyladenosine. Cell Res. (2017) 27:626–41. 10.1038/cr.2017.31 28281539 PMC5520850

[36] Zhang M Zhao K Xu X Yang Y Yan S Wei P. A peptide encoded by circular form of LINC-PINT suppresses oncogenic transcriptional elongation in glioblastoma. Nat. Commun. (2018) 9:1–17. 10.1038/s41467-018-06862-2 30367041 PMC6203777

[37] Zhang M Huang N Yang X Luo J Yan S Xiao F. A novel protein encoded by the circular form of the SHPRH gene suppresses glioma tumorigenesis. Oncogene. (2018) 37:1805–14. 10.1038/s41388-017-0019-9 29343848

[38] Ashwal-Fluss R Meyer M Pamudurti NR Ivanov A Bartok O Hanan M. circRNA biogenesis competes with pre-mRNA splicing. Mol. Cell. (2014) 56:55–66. 10.1016/j.molcel.2014.08.019 25242144

[39] Du WW Yang W Liu E Yang Z Dhaliwal P Yang BB. Foxo3 circular RNA retards cell cycle progression via forming ternary complexes with p21 and CDK2. Nucleic Acids Res. (2016) 44:2846–58. 10.1093/nar/gkw027 26861625 PMC4824104

[40] Zeng Y Du WW Wu Y Yang Z Awan FM Li X. A circular RNA binds to and activates AKT phosphorylation and nuclear localization reducing apoptosis and enhancing cardiac repair. Theranostics. (2017) 7:3842. 10.7150/thno.19764 29109781 PMC5667408

[41] Chen N Zhao G Yan X Lv Z Yin H Zhang S. A novel FLI1 exonic circular RNA promotes metastasis in breast cancer by coordinately regulating TET1 and DNMT1. Genome Biol. (2018) 19:1–14. 10.1186/s13059-018-1594-y 30537986 PMC6290540

[42] Anfossi S Babayan A Pantel K Calin GA. Clinical utility of circulating non-coding RNAs - an update. Nat. Rev. Clin. Oncol. (2018) 15:541–63. 10.1038/s41571-018-0035-x 29784926

[43] Chen YP Chan ATC Le QT Blanchard P Sun Y Ma J. Nasopharyngeal carcinoma. Lancet. (2019) 394:64–80. 10.1016/S0140-6736(19)30956-0 31178151

[44] Shuai M Hong J Huang D Zhang X Tian Y. Upregulation of circRNA_0000285 serves as a prognostic biomarker for nasopharyngeal carcinoma and is involved in radiosensitivity. Oncol. Lett. (2018) 16:6495–501. 10.3892/ol.2018.9471 30405788 PMC6202549

[45] Luo Y Ma J Liu F Guo J Gui R. Diagnostic value of exosomal circMYC in radioresistant nasopharyngeal carcinoma. Head Neck. (2020) 42:3702–11. 10.1002/hed.26441 32945062

[46] Wang J Kong J Nie Z Chen D Qiang J Gao W. Circular RNA Hsa_circ_0066755 as an oncogene via sponging miR-651 and as a promising diagnostic biomarker for nasopharyngeal carcinoma. Int. J. Med. Sci. (2020) 17:1499–507. 10.7150/ijms.47024 32669952 PMC7359393

[47] Wang Q Liu H Liu Z Yang L Zhou J Cao X. Circ-SLC7A5, a potential prognostic circulating biomarker for detection of ESCC. Cancer Genet. (2020) 240:33–9. 10.1016/j.cancergen.2019.11.001 31726270

[48] Collins LG Haines C Perkel R Enck RE. Lung cancer: diagnosis and management. Am. Fam. Physician. (2007) 75:56–63. 10.1186/1471-2296-8-1 17225705

[49] Li L Li W Chen N Zhao H Xu G Zhao Y. FLI1 exonic circular RNAs as a novel oncogenic driver to promote tumor metastasis in small cell lung cancer. Clin. Cancer Res. (2019) 25:1302–17. 10.1158/1078-0432.CCR-18-1447 30429198

[50] Hang D Zhou J Qin N Zhou W Ma H Jin G. A novel plasma circular RNA circFARSA is a potential biomarker for non-small cell lung cancer. Cancer Med. (2018) 7:2783–91. 10.1002/cam4.1514 29722168 PMC6010816

[51] Tan S Gou Q Pu W Guo C Yang Y Wu K. Circular RNA F-circEA produced from EML4-ALK fusion gene as a novel liquid biopsy biomarker for non-small cell lung cancer. Cell Res. (2018) 28:693–5. 10.1038/s41422-018-0033-7 29628502 PMC5993747

[52] Liu X-X Yang Y-E Liu X Zhang M-Y Li R Yin H-Y. A two-circular RNA signature as a noninvasive diagnostic biomarker for lung adenocarcinoma. J. Transl. Med. (2019) 17:50. 10.1186/s12967-019-1800-z 30777071 PMC6380039

[53] Wu SG He ZY Zhou J Sun JY Li FY Lin Q. Serum levels of CEA and CA15-3 in different molecular subtypes and prognostic value in Chinese breast cancer. Breast. (2014) 23:88–93. 10.1016/j.breast.2013.11.003 24291374

[54] Torre LA Bray F Siegel RL Ferlay J Lortet-Tieulent J Jemal A. Global cancer statistics, 2012. CA Cancer J. Clin. (2015) 65:87–108. 10.3322/caac.21262 25651787

[55] Li P Chen S Chen H Mo X Li T Shao Y. Using circular RNA as a novel type of biomarker in the screening of gastric cancer. Clin. Chim. Acta. (2015) 444:132–6. 10.1016/j.cca.2015.02.018 25689795

[56] Chen S Li T Zhao Q Xiao B Guo J. Using circular RNA hsa_circ_0000190 as a new biomarker in the diagnosis of gastric cancer. Clin. Chim. Acta. (2017) 466:167–71. 10.1016/j.cca.2017.01.025 28130019

[57] Zhao Q Chen S Li T Xiao B Zhang X. Clinical values of circular RNA 0000181 in the screening of gastric cancer. J. Clin. Lab Anal. (2018) 32:e22333. 10.1002/jcla.22333 28940688 PMC6817246

[58] Notarpaolo A Layese R Magistri P Gambato M Colledan M Magini G. Validation of the AFP model as a predictor of HCC recurrence in patients with viral hepatitis-related cirrhosis who had received a liver transplant for HCC. J. Hepatol. (2017) 66:552–9. 10.1016/j.jhep.2016.10.038 27899297

[59] Li J Gao T Gu S Zhi J Yang J Li G. An electrochemical biosensor for the assay of alpha-fetoprotein-L3 with practical applications. Biosens. Bioelectron. (2017) 87:352–7. 10.1016/j.bios.2016.08.071 27587360

[60] Lok AS Sterling RK Everhart JE Wright EC Hoefs JC Di Bisceglie AM. Des-gamma-carboxy prothrombin and alpha-fetoprotein as biomarkers for the early detection of hepatocellular carcinoma. Gastroenterology. (2010) 138:493–502. 10.1053/j.gastro.2009.10.031 19852963 PMC2819612

[61] Rawla P Sunkara T Gaduputi V. Epidemiology of pancreatic cancer: global trends, etiology and risk factors. World J. Oncol. (2019) 10:10–27. 10.14740/wjon1166 30834048 PMC6396775

[62] Siegel RL Miller KD Jemal A. Cancer statistics, 2020. CA Cancer J. Clin. (2020) 70:7–30. 10.3322/caac.21590 31912902

[63] McGuigan A Kelly P Turkington RC Jones C Coleman HG McCain RS. Pancreatic cancer: a review of clinical diagnosis, epidemiology, treatment and outcomes. World J. Gastroenterol. (2018) 24:4846–61. 10.3748/wjg.v24.i43.4846 30487695 PMC6250924

[64] Yang F Liu DY Guo JT Ge N Zhu P Liu X. Circular RNA circ-LDLRAD3 as a biomarker in diagnosis of pancreatic cancer. World J. Gastroenterol. (2017) 23:8345–54. 10.3748/wjg.v23.i47.8345 29307994 PMC5743505

[65] Li Z Yanfang W Li J Jiang P Peng T Chen K. Tumor-released exosomal circular RNA PDE8A promotes invasive growth via the miR-338/MACC1/MET pathway in pancreatic cancer. Cancer Lett. (2018) 432:237–50. 10.1016/j.canlet.2018.04.035 29709702

[66] Li J Li Z Jiang P Peng M Zhang X Chen K. Circular RNA IARS (circ-IARS) secreted by pancreatic cancer cells and located within exosomes regulates endothelial monolayer permeability to promote tumor metastasis. J. Exp. Clin. Cancer Res. (2018) 37:177. 10.1186/s13046-018-0822-3 30064461 PMC6069563

[67] Seimiya T Otsuka M Iwata T Tanaka E Sekiba K Shibata C. Aberrant expression of a novel circular RNA in pancreatic cancer. J. Hum. Genet. (2020) 66:181–91. 10.1038/s10038-020-00826-5 32879441

[68] Cho WC. Epigenetic alteration of microRNAs in feces of colorectal cancer and its clinical significance. Expert Rev. Mol. Diagn. (2011) 11:691–4. 10.1586/erm.11.57 21902530

[69] Zhang W Yang S Liu Y Wang Y Lin T Li Y. Hsa_circ_0007534 as a blood-based marker for the diagnosis of colorectal cancer and its prognostic value. Int. J. Clin. Exp. Pathol. (2018) 11:1399–406. 31938236 PMC6958175

[70] Ji W Qiu C Wang M Mao N Wu S Dai Y. Hsa_circ_0001649: a circular RNA and potential novel biomarker for colorectal cancer. Biochem. Biophys. Res. Commun. (2018) 497:122–6. 10.1016/j.bbrc.2018.02.036 29421663

[71] Tian J Xi X Wang J Yu J Huang Q Ma R. CircRNA hsa_circ_0004585 as a potential biomarker for colorectal cancer. Cancer Manage. Res. (2019) 11:5413–23. 10.2147/CMAR.S199436 31354349 PMC6573007

[72] Li XN Wang ZJ Ye CX Zhao BC Huang XX Yang L. Circular RNA circVAPA is up-regulated and exerts oncogenic properties by sponging miR-101 in colorectal cancer. Biomed. Pharmacother. (2019) 112:108611. 10.1016/j.biopha.2019.108611 30797148

[73] Pan B Qin J Liu X He B Wang X Pan Y. Identification of serum exosomal hsa-circ-0004771 as a novel diagnostic biomarker of colorectal cancer. Front. Genet. (2019) 10:1096. 10.3389/fgene.2019.01096 31737058 PMC6838203

[74] Yang N Xu B Kong P Han M Li BH. Hsa_circ_0002320: a novel clinical biomarker for colorectal cancer prognosis. Medicine. (2020) 99:e21224. 10.1097/MD.0000000000021224 32664176 PMC7360325

[75] Xu ZQ Yang MG Liu HJ Su CQ. Circular RNA hsa_circ_0003221 (circPTK2) promotes the proliferation and migration of bladder cancer cells. J. Cell Biochem. (2018) 119:3317–25. 10.1002/jcb.26492 29125888

[76] Pan J Xie X Li H Li Z Ren C Ming L. Detection of serum long non-coding RNA UCA1 and circular RNAs for the diagnosis of bladder cancer and prediction of recurrence. Int. J. Clin. Exp. Pathol. (2019) 12:2951–8. 31934131 PMC6949697

[77] Morice P Leary A Creutzberg C Abu-Rustum N Darai E. Endometrial cancer. Lancet. (2016) 387:1094–108. 10.1016/S0140-6736(15)00130-0 26354523

[78] Wang YM Huang LM Li DR Shao JH Xiong SL Wang CM. Hsa_circ_0101996 combined with hsa_circ_0101119 in peripheral whole blood can serve as the potential biomarkers for human cervical squamous cell carcinoma. Int. J. Clin. Exp. Pathol. (2017) 10:11924–31. 31966557 PMC6966017

[79] Tang X Liu S Ding Y Guo C Guo J Hua K. Serum circular FoxO3a serves as a novel prognostic biomarker in squamous cervical cancer. Cancer Manage. Res. (2020) 12:2531–40. 10.2147/CMAR.S243329 32308490 PMC7154007

[80] Hirajima S Komatsu S Ichikawa D Takeshita H Konishi H Shiozaki A. Clinical impact of circulating miR-18a in plasma of patients with oesophageal squamous cell carcinoma. Br. J. Cancer. (2013) 108:1822–9. 10.1038/bjc.2013.148 23579215 PMC3658511

[81] Huang E Fu J Yu Q Xie P Yang Z Ji H. CircRNA hsa_circ_0004771 promotes esophageal squamous cell cancer progression via miR-339-5p/CDC25A axis. Epigenomics. (2020) 12:587–603. 10.2217/epi-2019-0404 32050790

[82] Harris L Fritsche H Mennel R Norton L Ravdin P Taube S. American society of clinical oncology 2007 update of recommendations for the use of tumor markers in breast cancer. J. Clin. Oncol. (2007) 25:5287–312. 10.1200/JCO.2007.14.2364 17954709

[83] Yin WB Yan MG Fang X Guo JJ Xiong W Zhang RP. Circulating circular RNA hsa_circ_0001785 acts as a diagnostic biomarker for breast cancer detection. Clin. Chim. Acta. (2018) 487:363–8. 10.1016/j.cca.2017.10.011 29045858

[84] Nanishi K Konishi H Shoda K Arita T Kosuga T Komatsu S. Circulating circERBB2 as a potential prognostic biomarker for gastric cancer: an investigative study. Cancer Sci. (2020) 111:4177–86. 10.1111/cas.14645 32896032 PMC7648027

[85] Li WH Song YC Zhang H Zhou ZJ Xie X Zeng QN. Decreased expression of Hsa_circ_00001649 in gastric cancer and its clinical significance. Dis. Markers. (2017) 2017:4587698. 10.1155/2017/4587698 28167847 PMC5266807

[86] Huang M He YR Liang LC Huang Q Zhu ZQ. Circular RNA hsa_circ_0000745 may serve as a diagnostic marker for gastric cancer. World J. Gastroenterol. (2017) 23:6330–8. 10.3748/wjg.v23.i34.6330 28974900 PMC5603500

[87] Sun H Xi P Sun Z Wang Q Zhu B Zhou J. Circ-SFMBT2 promotes the proliferation of gastric cancer cells through sponging miR-182-5p to enhance CREB1 expression. Cancer Manage. Res. (2018) 10:5725–34. 10.2147/CMAR.S172592 30510446 PMC6248399

[88] Zhang X Zhou H Jing W Luo P Qiu S Liu X. The circular RNA hsa_circ_0001445 regulates the proliferation and migration of hepatocellular carcinoma and may serve as a diagnostic biomarker. Dis. Markers. (2018) 2018:3073467. 10.1155/2018/3073467 29785229 PMC5896272

[89] Zhang X Xu Y Qian Z Zheng W Wu Q Chen Y. circRNA_104075 stimulates YAP-dependent tumorigenesis through the regulation of HNF4a and may serve as a diagnostic marker in hepatocellular carcinoma. Cell Death Dis. (2018) 9:1091. 10.1038/s41419-018-1132-6 30361504 PMC6202383

[90] Wu C Deng L Zhuo H Chen X Tan Z Han S. Circulating circRNA predicting the occurrence of hepatocellular carcinoma in patients with HBV infection. J. Cell Mol. Med. (2020) 24:10216–22. 10.1111/jcmm.15635 32692470 PMC7520265

[91] Levin B Brooks D Smith RA Stone A. Emerging technologies in screening for colorectal cancer: CT colonography, immunochemical fecal occult blood tests, and stool screening using molecular markers. CA Cancer J. Clin. (2003) 53:44–55. 10.3322/canjclin.53.1.44 12568443

[92] Lin J Cai D Li W Yu T Mao H Jiang S. Plasma circular RNA panel acts as a novel diagnostic biomarker for colorectal cancer. Clin. Biochem. (2019) 74:60–68. 10.1016/j.clinbiochem.2019.10.012 31669510

[93] Ye DX Wang SS Huang Y Chi P. A 3-circular RNA signature as a noninvasive biomarker for diagnosis of colorectal cancer. Cancer Cell Int. (2019) 19:276. 10.1186/s12935-019-0995-7 31700498 PMC6829842

[94] Ehdaie B Atoria CL Lowrance WT Herr HW Bochner BH Donat SM. Adherence to surveillance guidelines after radical cystectomy: a population-based analysis. Urol. Oncol. (2014) 32:779–84. 10.1016/j.urolonc.2014.01.024 24935876

[95] Chi BJ Zhao DM Liu L Yin XZ Wang FF Bi S. Downregulation of hsa_circ_0000285 serves as a prognostic biomarker for bladder cancer and is involved in cisplatin resistance. Neoplasma. (2019) 66:197–202. 10.4149/neo_2018_180318N185 30509102

[96] Xu H Gong Z Shen Y Fang Y Zhong S. Circular RNA expression in extracellular vesicles isolated from serum of patients with endometrial cancer. Epigenomics. (2018) 10:187–97. 10.2217/epi-2017-0109 29334253

[97] Leonardi S Buttarelli M De Stefano I Ferrandina G Petrillo M Babini G. The relevance of prelamin A and RAD51 as molecular biomarkers in cervical cancer. Oncotarget. (2017) 8:94247–58. 10.18632/oncotarget.21686 29212225 PMC5706871

[98] Juliusson G Hough R. Leukemia. Prog. Tumor Res. (2016) 43:87–100. 10.1159/000447076 27595359

[99] Pan Y Lou J Wang H An N Chen H Zhang Q. CircBA9.3 supports the survival of leukaemic cells by up-regulating c-ABL1 or BCR-ABL1 protein levels. Blood Cells Mol. Dis. (2018) 73:38–44. 10.1016/j.bcmd.2018.09.002 30224298

[100] Feng XQ Nie SM Huang JX Li TL Zhou JJ Wang W. Circular RNA circHIPK3 serves as a prognostic marker to promote chronic myeloid leukemia progression. Neoplasma. (2020) 67:171–7. 10.4149/neo_2018_181129N908 31307197

[101] Long GV Hauschild A Santinami M Atkinson V Mandalà M Chiarion-Sileni V. Adjuvant dabrafenib plus trametinib in stage III BRAF-mutated melanoma. N. Engl. J. Med. (2017) 377:1813–23. 10.1056/NEJMoa1708539 28891408

[102] Yin D Wei G Yang F Sun X. Circular RNA has circ 0001591 promoted cell proliferation and metastasis of human melanoma via ROCK1/PI3K/AKT by targeting miR-431-5p. Hum. Exp. Toxicol. (2020) 40:310–24. 10.1177/0960327120950014 32830578

[103] Saraf AJ Fenger JM Roberts RD. Osteosarcoma: accelerating progress makes for a hopeful future. Front. Oncol. (2018) 8:4. 10.3389/fonc.2018.00004 29435436 PMC5790793

[104] Marais LC Bertie J Rodseth R Sartorius B Ferreira N. Pre-treatment serum lactate dehydrogenase and alkaline phosphatase as predictors of metastases in extremity osteosarcoma. J. Bone Oncol. (2015) 4:80–4. 10.1016/j.jbo.2015.09.002 26587373 PMC4648997

[105] Kun-Peng Z Chun-Lin Z Jian-Ping H Lei Z. A novel circulating hsa_circ_0081001 act as a potential biomarker for diagnosis and prognosis of osteosarcoma. Int. J. Biol. Sci. (2018) 14:1513–20. 10.7150/ijbs.27523 30263004 PMC6158732

[106] Zhu K Niu L Wang J Wang Y Zhou J Wang F. Circular RNA hsa_circ_0000885 levels are increased in tissue and serum samples from patients with osteosarcoma. Med. Sci. Monit. (2019) 25:1499–1505. 10.12659/MSM.914899 30802235 PMC6400018

[107] Li Y Zheng Q Bao C Li S Guo W Zhao J. Circular RNA is enriched and stable in exosomes: a promising biomarker for cancer diagnosis. Cell Res. (2015) 25:981–4. 10.1038/cr.2015.82 26138677 PMC4528056

[108] Jeck WR Sharpless NE. Detecting and characterizing circular RNAs. Nat. Biotechnol. (2014) 32:453–61. 10.1038/nbt.2890 24811520 PMC4121655

[109] Mariotto AB Yabroff KR Shao Y Feuer EJ Brown ML. Projections of the cost of cancer care in the United States: 2010-2020. J. Natl. Cancer Inst. (2011) 103:117–28. 10.1093/jnci/djq495 21228314 PMC3107566

[110] Keehan SP Cuckler GA Poisal JA Sisko AM Smith SD Madison AJ. National health expenditure projections, 2019-28: expected rebound in prices drives rising spending growth. Health Aff. (2020) 39:704–14. 10.1377/hlthaff.2020.00094 32207998

[111] Blumen H Fitch K Polkus V. Comparison of treatment costs for breast cancer, by tumor stage and type of service. Am. Health Drug Benefits. (2016) 9:23–32. 27066193 PMC4822976

[112] Kakushadze Z Raghubanshi R Yu W. Estimating cost savings from early cancer diagnosis. Data. (2017) 2:30. 10.3390/data2030030

[113] Okholm TLH Nielsen MM Hamilton MP Christensen LL Vang S Hedegaard J. Circular RNA expression is abundant and correlated to aggressiveness in early-stage bladder cancer. NPJ Genom. Med. (2017) 2:36. 10.1038/s41525-017-0038-z 29263845 PMC5705701

